# The putative mechanism of lymphopenia in COVID-19 patients

**DOI:** 10.1093/jmcb/mjac034

**Published:** 2022-05-31

**Authors:** Xinling Wang, Zezhong Liu, Lu Lu, Shibo Jiang

**Affiliations:** Key Laboratory of Medical Molecular Virology (MOE/NHC/CAMS), Institute of Infectious Disease and Biosecurity, School of Basic Medical Sciences, Fudan University, Shanghai 200032, China; Key Laboratory of Medical Molecular Virology (MOE/NHC/CAMS), Institute of Infectious Disease and Biosecurity, School of Basic Medical Sciences, Fudan University, Shanghai 200032, China; Key Laboratory of Medical Molecular Virology (MOE/NHC/CAMS), Institute of Infectious Disease and Biosecurity, School of Basic Medical Sciences, Fudan University, Shanghai 200032, China; Key Laboratory of Medical Molecular Virology (MOE/NHC/CAMS), Institute of Infectious Disease and Biosecurity, School of Basic Medical Sciences, Fudan University, Shanghai 200032, China; Department of Infectious Diseases and Shenzhen Key Lab of Endogenous Infection, Shenzhen Nanshan People's Hospital and the Sixth Affiliated Hospital of Guangdong Medical University, Shenzhen 518052, China

Severe acute respiratory syndrome coronavirus 2 (SARS-CoV-2) and its variants have caused >500 million confirmed cases and >6 million deaths. Apart from the common clinical manifestations, 63% of admitted coronavirus disease 2019 (COVID-19) patients had lymphopenia, increasing to 85% in patients with severe disease (Huang et al., 2020). It has been reported that several genes representing the p53-mediated apoptosis signaling pathway are upregulated in peripheral blood mononuclear cells (PBMCs) of COVID-19 patients (Xiong et al., 2020). Moreover, SARS-CoV-2 RNA is detected in most immune cells, including T and B lymphocytes and NK cells (Ren et al., 2021). Therefore, unraveling the mechanism of lymphopenia in COVID-19 patients depends on whether SARS-CoV-2 directly infects immune cells to induce cell death.

Previously, some researchers found SARS-CoV-2 protein and virus-like particles in CD4^+^ T lymphocytes, but no replication-competent viruses were produced *in vitro* (Banerjee et al., 2020). Some groups found the SARS-CoV-2 protein in monocytes but not in T, B, or NK cells (Zheng et al., 2021). Still, other investigators verified that SARS-CoV-2 could robustly infect activated T cells (Shen et al., 2022). Thus, this issue is still controversial.

**Figure 1:**
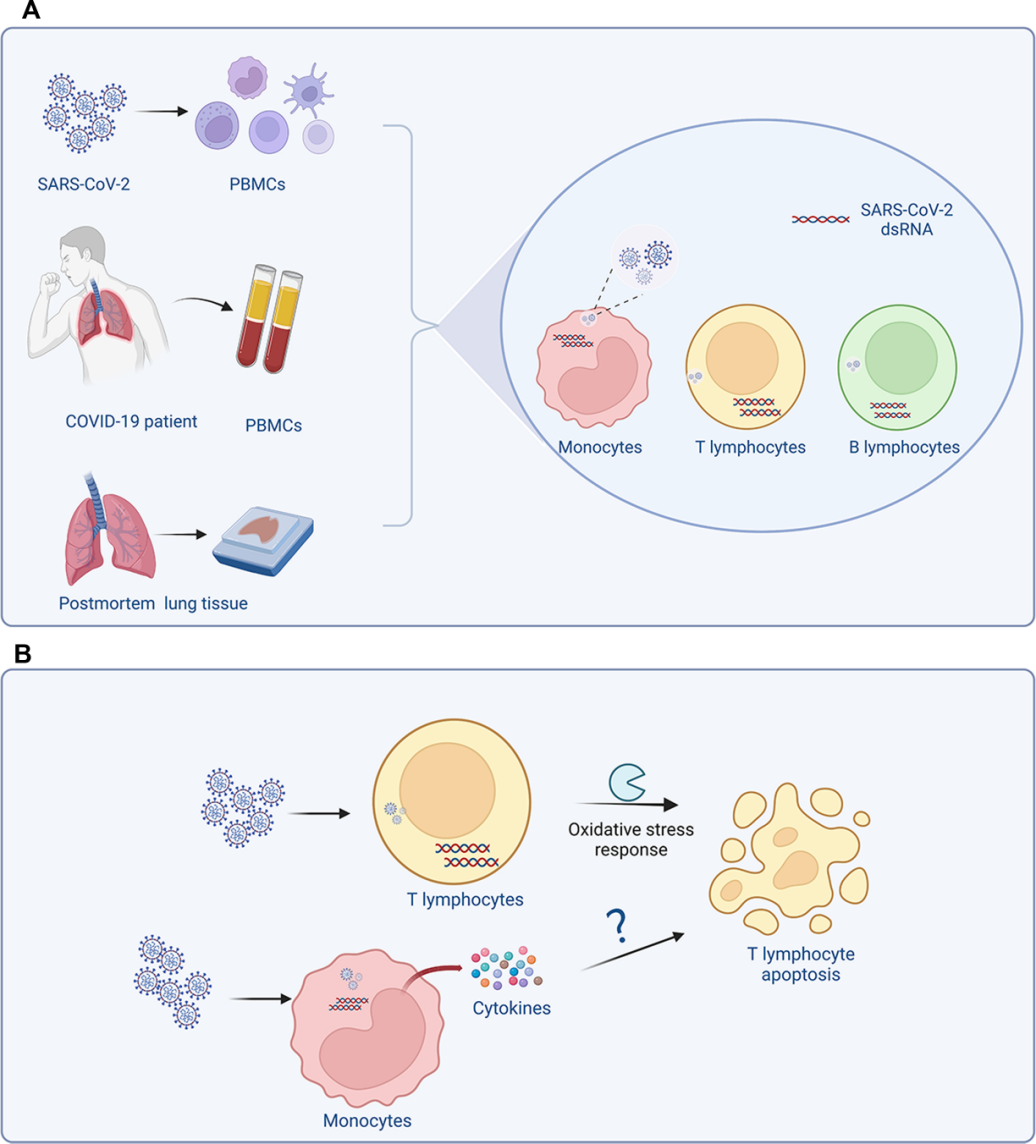
The putative mechanism of lymphopenia in COVID-19 patients. (**A**) Schematic representation of SARS-CoV-2 infection in human immune cells and its outcome. (**B**) The putative mechanism of T lymphocyte death. The figure was created with BioRender.com.

Using several biological experiments, Pontelli et al. (2022) showed that SARS-CoV-2 productively infects human PBMCs *in vitro*. However, viral titers peaked at 6–12 h post-infection and steadily decreased in the following hours. Nonetheless, double-stranded RNA was observed in monocytes, CD4^+^ and CD8^+^ T lymphocytes, and B lymphocytes, indicating that SARS-CoV-2 could infect and replicate in these cells. The authors detected high levels of active caspase 3/7, an apoptosis marker, in CD4^+^ T (49.2%) and CD8^+^ T (21%) lymphocytes but low levels in B lymphocytes (1.8%) and monocytes (1.1%). They also found that SARS-CoV-2 antigen was detected in 7.68% of the PBMCs from COVID-19 patients but not detected in all COVID-19 patients. In postmortem lung tissues from COVID-19 patients, SARS-CoV-2 antigen was detected in inflammatory monocytes, B lymphocytes, and CD4^+^ T lymphocytes, suggesting that these cells harbor SARS-CoV-2 infection (Figure 1).

Compared with other studies, some key points required special attention. First, Pontelli et al. (2022) discovered that antibodies against angiotensin-converting enzyme 2 (ACE2) could block SARS-CoV-2 infection of PBMCs, even though ACE2, the primary receptor for SARS-CoV-2 entry into cells, is expressed at extremely low levels in major PBMCs (Shen et al., 2022). To understand this inconsistency, LFA-1 and CD147 were confirmed as entry molecules able to mediate SARS-CoV-2 infection of T cells (Wang et al., 2020; Shen et al., 2022). The possible receptors mediating SARS-CoV-2 entry into other types of human immune cells are yet to be studied. Second, Pontelli et al. (2022) found that SARS-CoV-2 could infect and replicate in human immune cells. However, circulating monocytes fail to support the production of infectious SARS-CoV-2 progeny (Zheng et al., 2021; Junqueira et al., 2022). Moreover, it remains unknown whether SARS-CoV-2 infection of T cells is abortive (Banerjee et al., 2020) or productive (Shen et al., 2022). Thus, demonstrating the full profile of SARS-CoV-2-infected immune cells needs more evidence. Third, although Pontelli et al. (2022) considered that SARS-CoV-2 could infect B lymphocytes and monocytes directly, a recent study determined that SARS-CoV-2 infection of monocytes depended on antibody-mediated entry through Fcγ receptors (Junqueira et al., 2022). Owing to universal expression of the Fcγ receptor on the surface of monocytes and B cells, the formation of antibody-bound virus particles might be a pathway mediating viral entry into these cells *in vivo*.

SARS-CoV-2 infection of PBMCs *in vitro* could induce circulating T lymphocyte apoptosis, but only 6.7% of CD4^+^ and 2% of CD8^+^ T lymphocytes were concurrently positive for caspase 3/7 and icSARS-CoV-2. Thus, the authors considered that apoptosis induction occurs independently of viral replication in these cells. Given the high frequency of SARS-CoV-2-infected inflammatory monocytes, it is possible that some apoptotic T lymphocytes are induced by proinflammatory cytokines secreted by inflammatory monocytes. Previously, Shen et al. (2022) confirmed that the apoptosis of SARS-CoV-2-infected T lymphocytes (both unactivated and activated) from healthy donors and activated Jurkat T cells was induced by direct viral infection *in vitro*. Moreover, T cell death is probably dependent on mitochondrial ROS–hypoxia pathways (Shen et al., 2022). To sum up, cytokine storm and direct viral infection may be the cause for lymphopenia, but whether one or both of them drive T cell death *in vivo* still needs more studies to confirm (Figure 1).

In summary, Pontelli et al. (2022) found that SARS-CoV-2 directly infected and replicated in inflammatory monocytes and lymphocytes, leading to apoptosis of T lymphocytes *in vitro*. Moreover, SARS-CoV-2 was detected in the PMBCs and postmortem lung tissues of COVID-19 patients (Figure 1). These results shed light on the pathogenesis and progression of lymphopenia after SARS-CoV-2 infection within the host. However, more information is still needed to elucidate the pathogenic mechanisms of SARS-CoV-2 in human immune cells. Furthermore, the pathogenicity of emerging Omicron variants is significantly reduced (Shuai et al., 2022), but whether Omicron infection causes lymphopenia and whether lymphocytes are susceptible to Omicron are still unknown. Conducting related studies will provide certain clues for determining the pathogenic mechanisms of Omicron and other variants.
